# Magnetic resonance imaging evaluation of common peroneal nerve injury in acute and subacute posterolateral corner lesion: a retrospective study

**DOI:** 10.1590/0100-3984.2020.0072

**Published:** 2021

**Authors:** Gustavo Felix Marconi, Marcelo Novelino Simão, Fabricio Fogagnolo, Marcello Henrique Nogueira-Barbosa

**Affiliations:** 1 Faculdade de Medicina de Ribeirão Preto da Universidade de São Paulo (FMRP-USP), Ribeirão Preto, SP, Brazil.

**Keywords:** Peroneal nerve/diagnostic imaging, Knee injuries/diagnostic imaging, Knee/innervation, Magnetic resonance imaging, Nervo fibular/diagnóstico por imagem, Traumatismos do joelho/diagnóstico por imagem, Joelho/inervação, Ressonância magnética

## Abstract

**Objective:**

To evaluate qualitative and quantitative magnetic resonance imaging (MRI) criteria for injury of the common peroneal nerve (CPN) in patients with acute or subacute injuries in the posterolateral corner (PLC) of the knee, as well as to evaluate the reproducibility of MRI evaluation of CPN alterations.

**Materials and Methods:**

This was a retrospective study of 38 consecutive patients submitted to MRI and diagnosed with acute or subacute injury to the PLC of the knee (patient group) and 38 patients with normal MRI results (control group). Two musculoskeletal radiologists (designated radiologist A and radiologist B, respectively) evaluated the images. Nerve injury was classified as neurapraxia, axonotmesis, or neurotmesis. Signal strength was measured at the CPN, the tibial nerve (TN), and a superficial vein (SV). The CPN/TN and CPN/SV signal ratios were calculated. The status of each PLC structure, including the popliteal tendon, arcuate ligament, lateral collateral ligament, and biceps tendon, was classified as normal, partially torn, or completely torn, as was that of the cruciate ligaments. For the semiquantitative analysis of interobserver agreement, the kappa statistic was calculated, whereas a receiver operating characteristic (ROC) curve was used for the quantitative analysis.

**Results:**

In the patient group, radiologist A found CPN abnormalities in 15 cases (39.4%)-neurapraxia in eight and axonotmesis in seven-whereas radiologist B found CPN abnormalities in 14 (36.8%)-neurapraxia in nine and axonotmesis in five. The kappa statistic showed excellent interobserver agreement. In the control group, the CPN/TN signal ratio ranged from 0.63 to 1.1 and the CPN/SV signal ratio ranged from 0.16 to 0.41, compared with 1.30-4.02 and 0.27-1.08, respectively, in the patient group. The ROC curve analysis demonstrated that the CPN/TN signal ratio at a cutoff value of 1.39 had high (93.3%) specificity for the identification of nerve damage, compared with 81.3% for the CPN/SV signal ratio at a cutoff value of 0.41.

**Conclusion:**

CPN alterations are common in patients with PLC injury detected on MRI, and the level of interobserver agreement for such alterations was excellent. Calculating the CPN/TN and CPN/SV signal ratios may increase diagnostic confidence. We recommend systematic analysis of the CPN in cases of PLC injury.

## Abstract (pt)

**Objetivo:**

Avaliar critérios de identificação de lesão no nervo fibular comum (NFC) por ressonância magnética (RM) em pacientes com lesão aguda e subaguda no canto posterolateral (CPL) e avaliar a reprodutibilidade na identificação de alterações do NFC.

**Materiais e Métodos:**

Foram incluídos, retrospectivamente, 38 pacientes consecutivos submetidos a RM e com diagnóstico de lesão aguda ou subaguda no CPL do joelho, constituindo o grupo de pacientes. Outros 38 pacientes com RM normal foram utilizados como grupo controle. Dois radiologistas musculoesqueléticos (A e B) analisaram as imagens. A lesão neural foi classificada em neuropraxia, axoniotmese ou neurotmese. Foi medida a intensidade do sinal no NFC, no nervo tibial (NT) e em uma veia superficial (VS), e calculadas as razões de sinal NFC/NT e NFC/VS. Estruturas do CPL, incluindo tendão poplíteo, ligamento arqueado, ligamento colateral lateral e tendão do bíceps, foram classificadas como normal, rotura parcial ou rotura completa. Os ligamentos cruzados também foram avaliados segundo os mesmos critérios. O teste kappa foi utilizado para avaliar a concordância interobservador para as classificações semiquantitativas. Curva ROC foi utilizada para análise quantitativa.

**Resultados:**

O radiologista A encontrou alterações no NFC em 15 casos (39,4%): 8 neuropraxias, 7 axoniotmeses e nenhuma neurotmese. O radiologista B encontrou alterações no NFC em 14 casos (36,8%): 9 neuropraxias, 5 axoniotmeses e nenhuma neurotmese. O teste kappa mostrou alta concordância interobservador. No grupo controle, a razão NFC/NT variou entre 0,63-1,1 e a razão NFC/VS variou entre 0,16-0,41. Para o grupo de pacientes, a razão NFC/NT variou entre 1,30-4,02 e a razão NFC/VS variou entre 0,27-1,08. A análise com curva ROC demonstrou alta especificidade (93,3%) com relação à razão NFC/NT, usando um valor de corte de 1,39. Houve boa especificidade (81,3%) para a razão NFC/VS usando um valor de corte de 0,41.

**Conclusão:**

As alterações do NFC são detectadas com frequência nas lesões do CPL nas imagens de RM, com concordância interobservadores quase perfeita. Usando as razões de intensidade de sinal entre o NFC e o NT e entre o NFC e a VS, aumenta a confiança no diagnóstico. Recomendamos a análise sistemática do NFC nos casos de lesão do CPL.

## INTRODUCTION

Magnetic resonance imaging (MRI) is an excellent imaging method for diagnosing injuries to the musculoskeletal system and has been increasingly used for detecting peripheral nerve diseases. It is the imaging technique of choice for the diagnosis of traumatic injuries in the soft tissue of the knee, especially those involving the menisci, ligaments, muscles, or tendons. The posterolateral corner (PLC) is an anatomical complex located in the posterolateral region of the knee, being composed of myotendinous, ligamentous, and bone structures that promote biomechanical stability. These structures resist varus angulation, posterior translation, and external rotation^(1-3)^. The common peroneal nerve (CPN)-the lateral division of the sciatic nerve-has a curved, superficial course and presents a limited amount of epineurium, which makes it more susceptible to injuries^(4,5)^. Previous studies have demonstrated that the CPN and PLC are in close proximity, and that trauma can result in nerve damage in this region of the knee^(6-9)^.

Despite the fact that neural involvement may have future clinical implications, there have been few studies assessing the MRI findings of CPN abnormalities related to PLC injury. Therefore, the objective of this study was to evaluate the potential MRI findings of CPN abnormalities in patients with acute or subacute PLC lesion, seeking to identify correlations between damaged structures and nerve involvement, as well as to define MRI criteria for increasing accuracy in the diagnosis of nerve injury.

## MATERIALS AND METHODS

### Patients

This was a retrospective study of patients submitted to MRI at a university hospital between 2010 and 2013. There were 68 patients who met the initial eligibility criterion, which was having been diagnosed, on the basis of the MRI findings, with acute or subacute traumatic injury to the PLC caused by an isolated event or by an event in combination with a joint disorder, the event having occurred within the last 30 days before the examination. The study was approved by the research ethics committee of the institution. Because of the retrospective nature of the study, the requirement for written informed consent was waived. The examinations were tracked by searching the radiology information system of the institution for the terms “posterolateral corner”, “lateral collateral ligament”, “popliteus tendon”, “posterolateral capsule”, “arcuate ligament”, and “biceps femoris tendon”. The search process produced 47 patients who met these criteria. We excluded nine cases-four because the image quality was inadequate for CPN evaluation and five because the patients had previously undergone knee surgery. With the 38 remaining patients, we conducted a retrospective, cross-sectional case-control study.

The mean age of the patients was 31 years (range, 12-66 years). Of the 38 patients evaluated, 29 (76.3%) were male and 9 (23.6%) were female. Regarding the mechanism of trauma, 14 patients (36.8%) had suffered high-energy trauma (from motor vehicle accidents involving automobiles, motorcycles, pedestrians, or any combination of those), 16 patients (42.1%) had suffered low-energy trauma (sports injuries, falls from standing height, and sprains of various causes), and 8 patients (21.0%) had suffered trauma for which there was no detailed information regarding the mechanism.

A control group of 38 consecutive patients (selected from the radiology information system of same hospital) who underwent MRI examinations of the knee in which the results were reported as normal was used for comparative analysis in relation to the alterations found. The mean age of the patients in the control group was 32 years (range, 13-75 years). Of the 38 control group patients, 20 (52.6%) were male and 18 (47.4%) were female.

### MRI protocol

In 32 patients, the examinations were performed in a 1.5-T MRI scanner (Achieva; Koninklijke Philips N.V., Amsterdam, the Netherlands). In the remaining patients, the examinations were performed in a 3.0-T scanner (Discovery MR750w; GE Healthcare, Chicago, IL, USA). The clinical 1.5-T imaging protocol included a T1-weighted sequence (repetition time/echo time [TR/TE]: 532/10 ms); coronal, sagittal, and axial T2-weighted fast spin-echo sequences with fat saturation (TR/TE: 2635/60 ms); and a sagittal volumetric T2-weighted sequence with fat saturation (TR/TE: 2500/65 ms). For all 1.5-T sequences, we used a 16-cm field of view, a slice thickness of 4 mm, and a 176 × 220 matrix, except for the sagittal volumetric sequence, in which the slice thickness was 1.4 mm and the matrix was 176 × 220. The clinical 3.0-T imaging protocol included a T1-weighted sequence (TR/TE: 459/11 ms); and T2-weighted sequences, in all planes, with fat saturation (TR/TE: 1950-2000/60). For all 3.0-T sequences, we used an 18-cm field of view and a slice thickness of 4 mm; the matrix was 512 × 256 for the T1-weighted sequences and 384 × 256 for the T2-weighted sequences.

### Interpretation of images

Two musculoskeletal radiologists, working independently and blinded to the groups, performed the retrospective analysis of the MRI scans. One (radiologist A) had 15 years of experience in diagnostic imaging of the musculoskeletal system, and the other (radiologist B) was a clinical fellow in musculoskeletal radiology.

**Figure 1:**
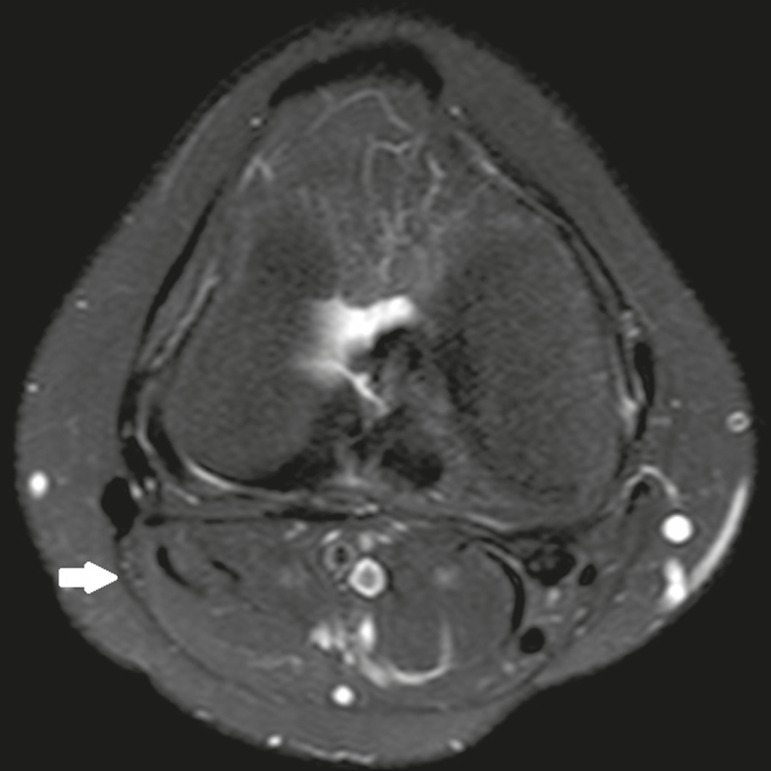
Axial T2-weighted MRI sequence with fat saturation, showing a normal CPN. The nerve presents normal signal intensity and a preserved fascicular pattern (arrow).

The CPN was analyzed regarding its trajectory, morphology, and signal intensity. The condition of the CPN was classified as normal, neurapraxic, axonotmetic, or neurotmetic, as proposed in the literature^(4,10,11)^. The criterion for classifying the nerve as normal was the absence of alteration of the signal and of the cross-sectional area (Figure 1).

*Neurapraxia*-Discrete lesion involving only the myelin sheath, in which MRI shows a hyperintense signal on T2-weighted and fluid-sensitive sequences and there may be a slight increase in the cross-sectional area.

**Figure 2:**
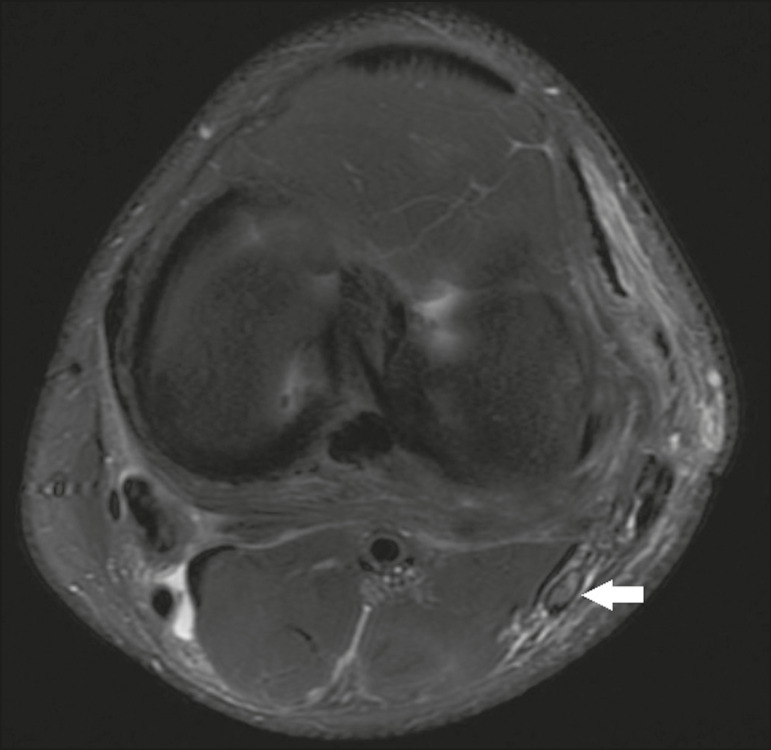
Axial T2-weighted MRI sequence with fat saturation, showing CPN axonotmesis. Note the increased signal intensity, clearly increased dimensions, and poorly defined fascicles in the CPN (arrow).

*Axonotmesis*-Axonal discontinuity and Wallerian degeneration in the distal segment, without involvement of the epineurium and the perineurium, in which MRI shows nerve thickening, together with poor definition and discontinuity of the nerve fascicles (Figure 2).

*Neurotmesis*-Severe form, with complete discontinuity of the nerve structure, in which MRI shows the discontinuity of the nerve and liquid or granulation tissue filling the gap between the stumps.

**Figure 3:**
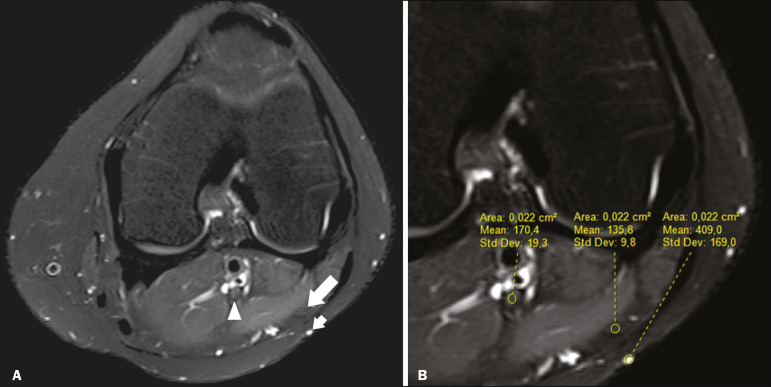
Signal intensity in the CPN and SV. A: Axial T2-weighted MRI sequence with fat saturation of a normal knee, showing the CPN (long arrow), TN (arrowhead), and SV (short arrow). B: The same image magnified for signal measurement in the area of interest. In this case, the CPN/TN and CPN/SV signal ratios were 0.79 and 0.33, respectively.

Signal intensity was also measured within the CPN, the tibial nerve (TN), and the superficial vein (SV) near the CPN, with specific software for viewing files in the Digital Imaging and Communication in Medicine format (Clear Canvas Workstation; Synaptive Medical Inc., Ontario, Canada), as shown in Figure 3. The images were magnified in a standardized manner to avoid the partial volume effect from surrounding tissues during measurements, and the CPN/TN and CPN/SV signal ratios were calculated. The CPN signal was measured at the point where it subjectively presented the highest signal intensity in the image, and the TN and SV signals were measured in the same image. When the SV was not sufficient for signal measurement at the level of the slice analyzed, the signal was measured in the previous or subsequent image. The analysis was conducted in the same manner for the control group cases.

The two radiologists, working independently, performed a systemic analysis of the following PLC structures to identify possible injuries: the myotendinous junction of the popliteus muscle; the arcuate ligament; the posterolateral capsule; the lateral collateral ligament; and the biceps femoris tendon. In view of the fact that the arcuate ligament constitutes a thickening of the posterolateral capsule and may be inconstant^(12)^, we considered these two structures separately for the purpose of the analysis. Each radiologist analyzed the anterior cruciate ligament and posterior cruciate ligament separately. All of these structures were classified as normal, partially torn, or completely torn. A tendon or ligament was defined as normal when its physiological orientation, signal intensity, and thickness were preserved. The criteria for a partial tear included signal alteration, thickening, thinning, or rupture of part of the ligament or tendon. The criteria used in order to define a complete tear included complete, unequivocal discontinuity, with a liquid signal in the resulting focal defect. Finally, the fibular head was classified as follows: normal; showing bone edema without a fracture line; showing a nondisplaced fracture; and showing a displaced fracture. To allow the evaluation of intraobserver agreement, radiologist B reanalyzed the images three months after the initial interpretation.

### Statistical analysis

To evaluate interobserver and intraobserver agreement for the classification of CPN lesions and PLC structures, we calculated the kappa statistic (κ). The results were interpreted as previously described^(12)^: no agreement (κ < 0); slight agreement (κ = 0-0.19); fair agreement (κ = 0.20-0.39); moderate agreement (κ = 0.40-0.59); substantial agreement (κ = 0.60-0.79); and excellent agreement (κ = 080-1.00).

To determine whether nerve injury correlated with specific lesions of the individual ligamentous and tendinous structures, we calculated odds ratios (ORs) and relative risk (RR). Spearman’s correlation coefficient was utilized to evaluate the relationship between the total number of ligamentous or tendinous structures involved and the nerve injury. We classified the lesions as grade 1 when only one ligamentous/tendinous structure was affected, grade 2 when two structures were affected, and so on.

We compared the group with PLC injury and the control group in terms of the CPN/TN and CPN/SV signal ratios, to assess any statistical difference between the groups. Finally, we constructed a receiver operating characteristic (ROC) curve to identify the best cutoff to differentiate between the two groups.

## RESULTS

**Figure 4:**
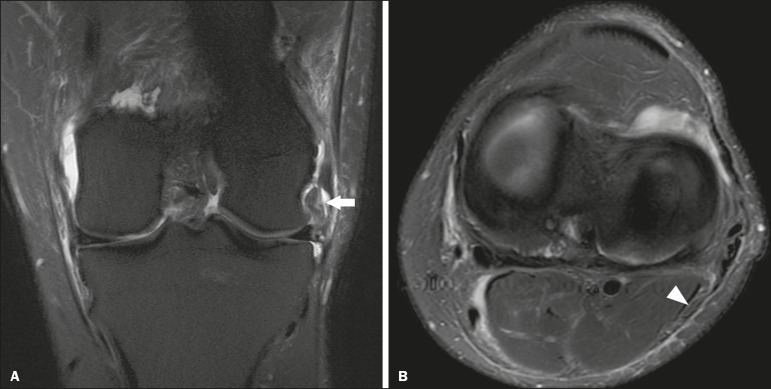
A 43-year-old male patient who suffered trauma playing soccer. **A:** Coronal T2-weighted MRI sequence with fat saturation, showing PLC lesion, highlighting the tear in the popliteus tendon (arrow). There was also injury to the joint capsule and the LCL (not shown). **B:** Axial T2-weighted MRI sequence with fat saturation, showing the CPN (arrow), which was considered normal in this case.

In the group of patients with PLC injury (n = 38), radiologist A identified CPN abnormalities in 14 cases (36.8%)-neurapraxia in 9 and axonotmesis in 5-whereas radiologist B identified CPN abnormalities in 15 (39.4%)-neurapraxia in 8 and axonotmesis in 7. The findings in the ligaments and tendons (Figure 4) are shown in Table 1. Regarding the classification of fibular alterations, radiologist A diagnosed 29 cases (76.3%) as normal, 8 cases (21.0%) with bone edema, and 1 case (2.6%) with a nondisplaced fracture, whereas radiologist B diagnosed 29 cases (76.3%) as normal, 7 (18.4%) with bone edema, 1 (2.6%) with a nondisplaced fracture, and 1 (2.6%) with a displaced fracture.

**Table 1:** MRI findings in tendinous and ligamentous structures.

Status	Anterior cruciate ligament	Posterior cruciate ligament	Lateral collateral ligament	Popliteus tendon	Biceps femoris tendon	Arcuate ligament
Normal						
Radiologist A	6 (15%)	19 (50%)	0 (0%)	9 (23%)	10 (26%)	9 (23%)
Radiologist B	7 (18%)	18 (47%)	1 (2%)	8 (21%)	15 (39%)	7 (18%)
Partial tear						
Radiologist A	11 (28%)	7 (18%)	21 (55%)	25 (65%)	17 (44%)	25 (65%)
Radiologist B	9 (23%)	9 (23%)	18 (47%)	24 (63%)	12 (31%)	25 (65%)
Complete tear						
Radiologist A	21 (55%)	12 (31%)	17 (44%)	4 (10%)	11 (28%)	4 (10%)
Radiologist B	22 (57%)	11 (28%)	19 (50%)	6 (15%)	11 (28%)	9 (23%)

There was excellent interobserver agreement (κ = 0.85) for the detection of CPN injury. The interobserver agreement was also excellent for the findings in the anterior cruciate ligament, posterior cruciate ligament, lateral collateral ligament, and popliteal tendon, the level of agreement ranging from κ = 0.81 (for the anterior cruciate ligament) to κ = 0.91 (for the posterior cruciate ligament). For the findings in the biceps femoris tendon and arcuate ligament, the interobserver agreement was substantial (κ = 0.72 and κ = 0.79, respectively). For each association, we calculated the 95% confidence interval.

There was excellent intraobserver agreement for the analysis of the CPN, anterior cruciate ligament, and lateral collateral ligament, the level of agreement ranging from κ = 0.84 (for the CPN) and κ = 0.91 (for the lateral collateral ligament). Agreement was substantial for the posterior cruciate ligament, biceps femoris tendon and arcuate ligament, the level of agreement ranging from κ = 0.62 and κ = 0.74. The level of agreement for the popliteus tendon was also substantial (κ = 0.60).

When we calculated the RR and OR to determine whether CPN injury was associated with an alteration in any individual tendinous and ligamentous structure, we found no statistical association. Likewise, we found no statistically significant correlation between the total number of affected ligamentous or tendinous structures and the presence of CPN injury (r = 0.31; *p* = 0.05). However, there was a trend toward an increase in the number of affected structures being accompanied by a increase in the degree of nerve damage.

Finally, we also compared the CPN/TN and CPN/SV signal ratios between the group of patients and the control group, using the ROC curve for quantitative analysis. The results of the ROC curve analysis (for radiologist A) are shown in Table 2.

**Table 2:** Cutoff, specificity, and sensitivity for the detection of nerve damage, in comparison with the control group.

Measure	Radiologist A	
	CPN/TN signal ratio	CPN/SV signal ratio
*Cut-off*	1.27	0.40
Specificity	98.8%	89.9%
Sensitivity	88.1%	71.6%

## DISCUSSION

The anatomy of the PLC has been described in various studies and review articles^(1-3,13-16)^, and it was not the objective of the present study to detail the region. There is divergence in the literature about the structures of the PLC. Seebacher et al.^(17)^ divided the region into three layers: superficial, middle, and deep. In subsequent studies^(1,18)^, other authors proposed changes in this definition, which have altered the description of the layers and affords a more standardized, systematic approach. The deep layer is the most important layer in biomechanical stabilization and is the layer that presents the greatest anatomical variability. Non-recognition of PLC lesions is a potential cause of persistent instability, failure of cruciate ligament grafts, and osteoarthritis^(18-24)^.

The CPN is the lateral branch of the sciatic nerve. It courses from the posterolateral side of the knee, around the biceps femoris tendon and fibular head, dividing, usually at the fibular neck, into three branches: the deep peroneal nerve; the superficial peroneal nerve; and the articular or recurrent branch. The deep peroneal nerve innervates the muscles of the anterior compartment of the leg (tibialis anterior, extensor digitorum longus, extensor hallucis longus, and peroneus tertius). The superficial peroneal nerve innervates the lateral compartment (short and long peroneal muscles) and provides sensation to the anterolateral lower leg. The recurrent branch provides sensory information to the proximal tibiofibular joint^(5,25,26)^.

**Figure 5:**
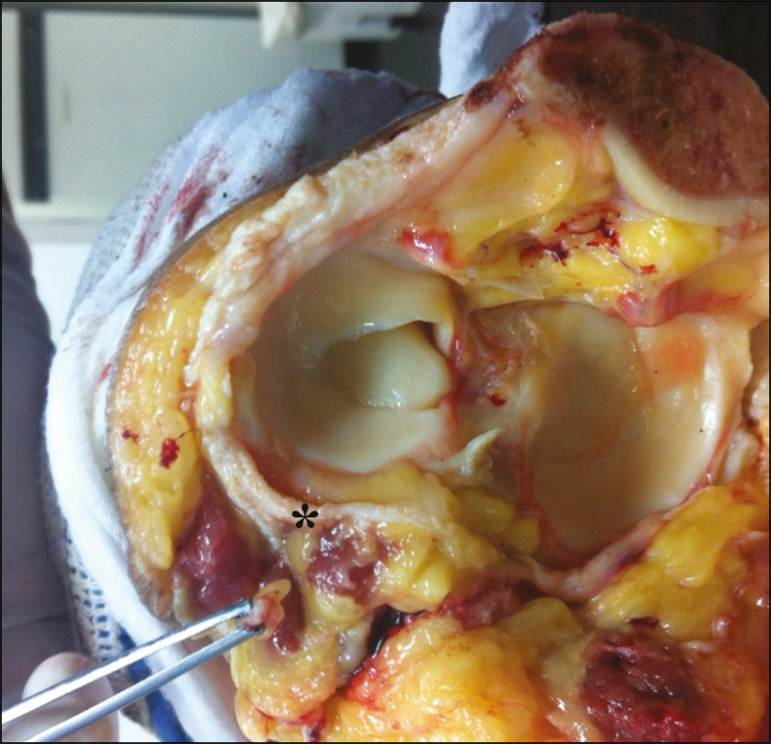
Cadaveric specimen of femorotibial joint prepared to show the anatomical relationships of the CPN. The CPN is grasped with a rat-tooth forceps. Note the proximity of the CPN to the posterior joint capsule (asterisk).

Because of anatomical features such as its superficiality, its trajectory, and the scarce amount of epineurium, the CPN and its branches are susceptible to lesions, traumatic or otherwise^(6-9,27-35)^. Studies have demonstrated that knee injuries and dislocations are associated with peroneal nerve lesion^(6-9,27-35)^. Trappeniers et al.^(9)^ reported three cases of post-trauma CPN injury in the PLC. Jia et al.^(7)^ demonstrated the anatomical proximity between the CPN and the PLC, where the distance between the nerve and the PLC structures can be 8 mm. The proximity between the CPN and PLC is illustrated in Figure 5. Bottomley et al.^(28)^ identified displacement of the CPN in PLC injuries, especially when there is distal avulsion of the biceps femoris tendon, and the surgeon should be aware of this possibility to avoid nerve damage during the surgical procedure.

**Figure 6:**
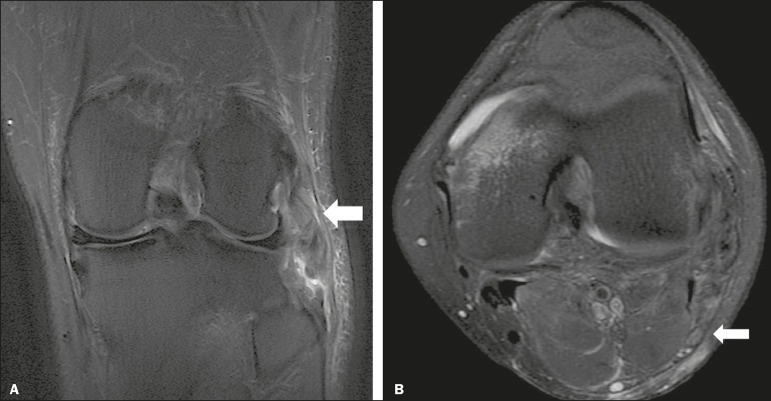
A 21-year-old male patient who was in a motorcycle accident. **A:** Coronal T2-weighted MRI sequence with fat saturation, showing injuries to PLC structures (arrow). Note the complete tears in the LCL and biceps femoris tendon, as well as the partial tears in the popliteal tendon and arcuate ligament. **B:** Axial T2-weighted sequence with fat saturation, showing the CPN with signal alteration but without discontinuities (arrow), which was interpreted as axonotmesis.

A number of recent studies conducted in Brazil have highlighted the important role that imaging methods play in the evaluation of the musculoskeletal system^(36-40)^. In our study, the prevalence of imaging alterations in the CPN was high (36.8-39.4%), and the levels of interobserver and intraobserver agreement for these abnormalities were excellent (Figure 6). The great majority of nerve damage that we identified by MRI was classified as neuropraxia, which is characterized by mild, transient lesions that have a favorable prognosis. Because of the retrospective nature of the study, we had difficulty in obtaining clinical information about the patients, which made it impossible, in general, to determine whether patients presented alterations of sensitivity or motor skills, such as paresthesia on the lateral face of the leg or difficulty in foot dorsiflexion. We believe that discrete nerve lesions, such as neuropraxia, may be asymptomatic or oligosymptomatic and therefore were not adequately reported in the clinical records. Only two patients were reported to have functional nerve damage, with paresthesia and foot drop. In both of those patients, the CPN was classified on MRI as axonotmetic, and the CPN/TN and CPN/SV signal ratios were clearly altered. Some patients presented multiple, complex damage, involving other systems, which may have hindered the evaluation of the peripheral neurological conditions in question.

We did not detect a statistical association between CPN injury and a tear in a specific ligamentous or tendinous structure. In isolation, none of the lesions of the PLC anatomical structures analyzed increased or decreased the risk for CPN injury. However, when analyzing the structures in conjunction (i.e., more than one affected structure), we detected a trend toward an increased risk of nerve damage.

Considering the classification of peripheral nerve injury performed by radiologist A as the reference, we found that the CPN/TN and CPN/SV signal ratios showed high sensitivity and specificity for nerve damage and, in this sense, have the potential to assist a less experienced radiologist in this diagnostic decision-making process. Using a cutoff of 1.27, we found the specificity and sensitivity of the CPN/TN signal ratio to be 98.8% and 88.1%, respectively, compared with 89.9% and 71.6%, respectively, for the CPN/SV signal ratio when we used a cutoff of 0.40. We suggest these ratios be used, especially the CPN/TN signal ratio, which is a useful tool for detecting MRI alteration in the CPN, increasing diagnostic certainty. The signal relationship between the injured nerve and an adjacent vein was previously used by Chhabra et al.^(41)^ who found that it showed high accuracy for the detection of neuropathy of the sciatic nerve. In the present study, we further added the CPN/TN signal ratio, which also proved to be a good instrument for increasing accuracy and diagnostic certainty.

Our study has some limitations. The greatest limitation is related to the retrospective nature of the study, which prevented us from obtaining reliable information regarding the clinical presentation of patients or regarding the clinical and imaging evolution, which could have implications for abnormalities seen on imaging in the acute and subacute phases. In a previous, prospective longitudinal study of a sample of patients^(42)^ with an epidemiological profile similar to that of the patient sample evaluated in the present study, five patients with chronic injury to the PLC, which was initially identified by MRI, were followed by MRI and by detailed physical examination. The authors found that chronic injuries to the CPN initially classified as neuropraxia by MRI had little or no effect on the clinical repercussion in evolution, whereas those classified as axonotmesis resulted in sensitivity/motor disorders. Another limitation is related to the presence of soft-tissue edema in cases of traumatic injury to the PLC, which precluded the complete blinding of the radiologists who performed the evaluations as to which group the patient belonged. In addition, we had no surgical data available, especially with regard to CPN status.

## CONCLUSION

We observed a high prevalence of CPN alterations in patients previously diagnosed with PLC injury detected on MRI, and the level of interobserver and intraobserver agreement for such observations was excellent. We suggest using the CPN/TN and CPN/SV signal ratios to increase diagnostic certainty. We also recommend careful analysis of the CPN in cases of PLC injury.
